# Universality of fold-encoded localized vibrations in enzymes

**DOI:** 10.1038/s41598-019-48905-8

**Published:** 2019-09-06

**Authors:** Yann Chalopin, Francesco Piazza, Svitlana Mayboroda, Claude Weisbuch, Marcel Filoche

**Affiliations:** 1 Laboratoire d’Energétique Macroscopique et Moléculaire, Combustion (EM2C), CentraleSupélec, CNRS, 91190 Gif-sur-Yvette, France; 2 Centre de Biophysique Moléculaire (CBM) CNRS UPR4301 & Université d’Orléans, Orléans, 45071 France; 3 School of Mathematics, University of Minnesota, Minneapolis, Minnesota 55455 USA; 4 Laboratoire de Physique de la Matière Condensée, Ecole Polytechnique, CNRS, 91128 Palaiseau, France; 5 Materials Department, University of California, Santa Barbara, California, 93106 USA

**Keywords:** Biological physics, Catalytic mechanisms

## Abstract

Enzymes speed up biochemical reactions at the core of life by as much as 15 orders of magnitude. Yet, despite considerable advances, the fine dynamical determinants at the microscopic level of their catalytic proficiency are still elusive. In this work, we use a powerful mathematical approach to show that rate-promoting vibrations in the picosecond range, specifically encoded in the 3D protein structure, are *localized* vibrations optimally coupled to the chemical reaction coordinates at the active site. Remarkably, our theory also exposes an hithertho unknown deep connection between the unique localization fingerprint and a distinct partition of the 3D fold into independent, foldspanning subdomains that govern long-range communication. The universality of these features is demonstrated on a pool of more than 900 enzyme structures, comprising a total of more than 10,000 experimentally annotated catalytic sites. Our theory provides a unified microscopic rationale for the subtle structure-dynamics-function link in proteins.

## Introduction

The intricate networks of metabolic cascades that power living organisms ultimately rest on the exquisite ability of enzymes to increase the rate of chemical reactions by many orders of magnitude. However, despite a large body of evidence accumulated over the past two decades in favor of the highly dynamical nature of proteins, the question whether protein motions such as conformational changes and finer (and faster) reorganization dynamics play a role in enzyme catalysis remains widely debated^1^.

Although many molecular machines contain intrinsically disordered domains^2^, the 3D fold is central to enzyme functioning. In particular, increasing evidence is accumulating in the literature in favor of the existence of specific fold-encoded motions believed to govern the relevant collective coordinate(s) that are coupled to the chemical tranformation step^1,3–8^. These motions typically correspond to localized vibrations of the protein scaffold that contribute to the catalytic reaction, i.e., modes that, if impeded, would lead to a deterioration of the catalytic efficiency^1^. The existence and importance of such localized, shape-specific motions, coined rate-promoting vibrations^6^ is backed by many computational and experimental studies^4,9–15^, beginning with the pioneering ideas by McClare on the functional role of non-equilibrium localized motions in muscle contraction^16^. The role of rate-promoting vibrations in enzymes has been highlighted for the tunneling reaction coordinate (i.e. the donor-acceptor distance) in lactate dehydrogenase (LDH)^17–19^. Promoting modes in Purine Nucleosidase phosphorylase have also been explored more recently^20^. Interestingly, evidence for the existence of promoting vibrations coupling directly to the reaction coordinate in enzyme-catalyzed hydrogen transfer reactions has also been gathered from the temperature dependence of kinetic isotope effect^21^. More generally, the key rate-promoting role of fluctuations in the region of the active site has been established on rigorous quantum mechanical grounds in the 1990s by Bruno and Bialek for enzymatic hydrogen transfer^22^. Yet, despite the broad set of evidence for specific dynamical effects in enzymes-catalyzed reactions, a universal demonstration of the existence of rate-promoting motions in enzymes that could explain how specific vibrations at the active site contribute to increase the reaction rate is still lacking.

To tackle the problem of assessing the role of vibrations in the catalytic efficiency of enzymes, it is essential to understand that in general protein motions play a rather diverse and subtle role over a wide range of timescale and distances^23^. The longest times, which correspond to conformational changes of the protein, are in the ms-s range^24^ and are generally believed not to be directly coupled to the enzymatic catalytic step, as most enzymes have turnover rates in the 10^3^ s^−1^ range^1^. The matter is subtler for allosteric transitions (i.e., action at a *distance*)^25^, and slow conformational sampling, occurring in the ms-s timescale too, with many studies advocating a variable degree of coupling of those motions to the chemical step^26,27^, including the key advances brought about by single-molecule enzymology^28,29^. Faster conformational sampling in the ns-ms and faster reorganization motions of the active sites in the ps-ns range are commonly accepted to play an important role in shaping the kinetic behavior of many enzymes, such as alcohol dehydrogenase^30^ and methylamine dehydrogenase^31^, as most clearly revealed by the pioneering studies on the role of protein motions in hydrogen tunneling in soybean lypoxygenase-1^30,32^.

Quantum-mechanical tunneling in hydrogen transfer at room temperature was first demonstrated in 1989 in a seminal paper on alcohol dehydrogenase^33^. In particular, this discovery revealed the tremendous power of kinetic isotope effects studies to investigate the direct coupling of fast vibrational modes localized at the active site to the catalytic step^34^. The general surprising finding is that the kinetic isotope effect is largely temperature-independent in many native enzyme systems^9,31,32^. This is usually interpreted as the blueprint of an optimal structural *compactness* at the active site, where reaction partners are kept tight in the optimal geometry that underlies the catalytically competent atomic arrangement. This fact perfectly rhymes with the known reports that active sites tend to lie in the stiffest regions of enzyme structures^35–37^ and that a subtle balance of rigidity and some specific flexibility are implied in enzyme catalysis^38,39^.

Taken together, the above facts lead to an emerging picture where enzymes feature highly compact, pre-organized active sites. These represent structurally competent catalytic precursors that are generically modulated through slow conformational sampling at the level of the whole structure, but more finely and specifically regulated by specific rate-promoting vibrations that couple directly to the reaction coordinate(s). Hydrogen tunneling kinetics provides the perfect grounds for illustrating these ideas. There is now a wide consensus that donor-acceptor distances at the active site for enzymes that catalyze the transfer of some hydrogen species are modulated with sampling frequencies in the 50–300 cm^−1^ range (1.5–9 THz)^34^, corresponding to motions in the ps-ns range. These rate-promoting vibrations provide optimal compression along the donor-acceptor separation, thus enhancing the tunneling rate through a vibrationally assisted mechanism^22,34^. In other words, fast conformational sampling along the donor-acceptor distance is optimal in the substrate-bound conformation, which generates active-site compression leading to favorable close approach between donor and acceptor atoms^34^ on timescales slower than tunneling times (fs).

In this paper, we take one step forward and show that fold-specific, localized vibrations enforcing dynamical compression at the active site are a universal feature of enzymes. This suggests that enzyme structures have evolved as optimally designed *mechanical transducers* of vibrational energy mediated by rate-promoting patterns^11^. The article is organized as follows: first, we introduce the localization landscape (LL), a novel and powerful mathematical tool which we use here to predict the spatial distribution of energy in proteins modeled by the Elastic Network Model (ENM)^40^. The implementation of the LL is illustrated for a specific example, the well-documented case of LDH, before reporting the results of a systematic study of the correlation between active sites and localized vibrations on a sample of about 1,000 enzymes (corresponding to more than 10,000 annotated actives sites).

## Materials and Methods

**Figure 1:**
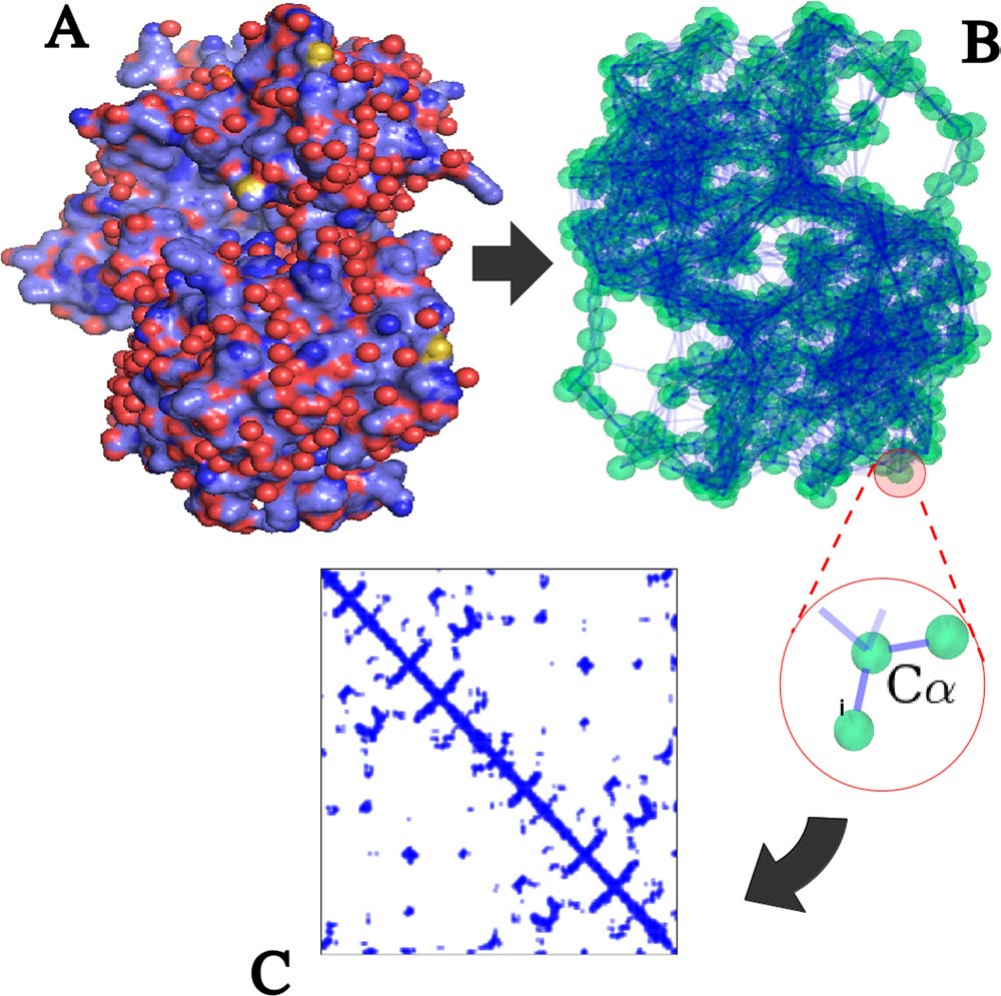
Coarse grained model. (**A**) All-atom view of the L-Lactate Dehydrogenase dimer (LDH, PDB id: 1I0Z). (**B**) Elastic Network Model deduced from the tertiary structure. (**C**) Sparsity pattern of the 3*N* × 3*N* force constant matrix ${{\mathbb{H}}}_{ij}^{\alpha \beta }$ used to compute the localization landscape. Each non-vanishing term is represented by a blue spot. In our case of uniform spring constant and sharp cutoff coupling, this matrix is a direct representation of the connectivity pattern among residues.

In order to investigate the structural origin of vibrational modes related to the active-site reorganization in the specific timescale of interest (100 cm^−1^, 3 THz), we adopt a coarse-grained elastic-network model (ENM)^36,40,41^ (see Supplementary Information). This model reduces each protein to a collection of beads and springs that interact according to a unique, fold-encoded connectivity pattern. In our case, the beads correspond to the amino acids centered at the *C*_*α*_ carbon of the tertiary structure (Fig. 1A). Enzymes are therefore seen as a set of coupled harmonic oscillators (Fig. 1B). As it is well known^42^, the local connectivity of each each amino acid is reflected in the sparsity pattern of the force constant matrix (Fig. 1C). This connectivity controls the localization pattern of high-frequency modes (${\mathscr{O}}{\mathrm{(10}}^{2})$ cm^−1^, i.e. about 3 THz, in *C*_*α*_-based ENM schemes).

The main idea of this paper is to use a novel mathematical tool, coined localization landscape (LL), to decipher the subtle structure-dynamics-function relation in enzymes. The LL, which rests on a universal theory of wave localization, unveils the localization pattern of standing waves in complex or disordered media^43^ and is extended here to the case of protein vibrations (see Supplementary Information). In this paper, bypassing the need to compute the full set of normal modes, the LL is defined at each site of the ENM network as the modulus of a real-valued vector. This vector field on the network is solution to a simple linear system based on the force constant matrix (see Supplementary Information). The LL provides the essential information about the interplay between the complex protein shape and the pattern of microscopic vibrations. In particular, the “valleys” of the LL delineate the main regions of existence of the large-amplitude localized vibrations, thus yielding an effective functional partition of the molecule structure. In addition, the local maxima of the LL identify the most localized vibrating areas or “hot spots”, while the corresponding values of the LL at these hot spots are very good predictors of the associated vibration frequencies^44,45^ (see also the Supplementary Information and more specifically Fig. 1). We emphasize here that the computation of the LL is about 50 times faster than solving the full eigenvalue problem (see Table I in Supplementary Information).

The LL reveals that the molecular architecture of enzymes seems so designed as to concentrate high-frequency vibrations on very small sets of specific residues, as it has been pointed out in previous studies^35,37,46^. Moreover, the LL offers considerable new insight into how the localization pattern also segments the molecular scaffold into nearly vibrationally independent (i.e., uncoupled) clusters of amino acids. Although this work focuses on enzymes, the localization property seems to remain a general feature of proteins.

## Results

**Figure 2:**
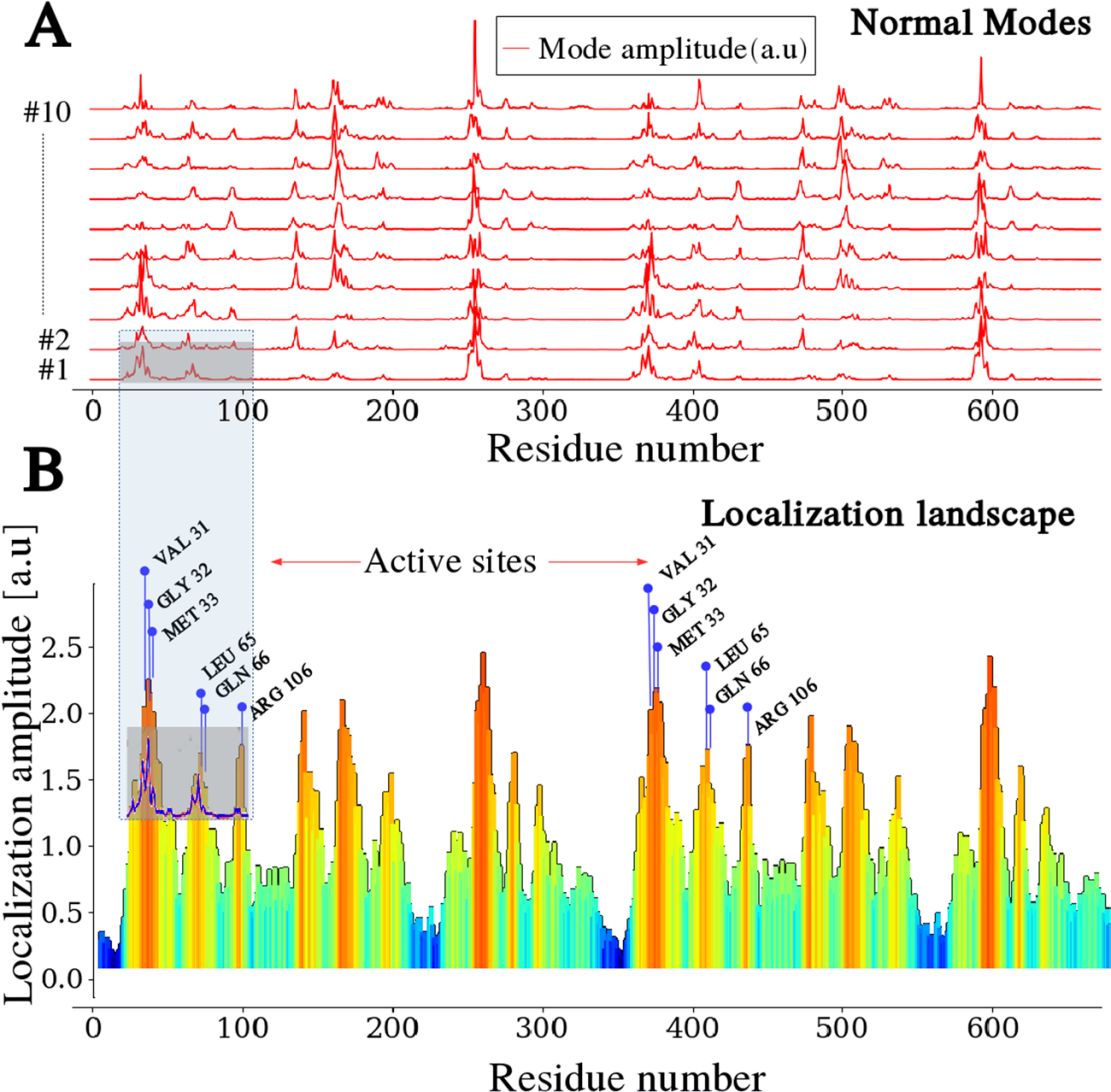
Comparison between Normal Modes and LL computation for human L-Lactate Dehydrogenase dimer (LDH, PDB id: 1I0Z). (**A**) Wave localization is visualized by plotting the 10 highest-frequency normal modes. Their frequencies range from 94.2 to 99.3 cm^−1^ (2.82–2.98 THz). (**B**) The LL (u) is drawn along the protein backbone. Catalytic sites, shown explicitly alongside the LL, clearly lie very close to the LL maxima, corresponding to the sites of highest localization (hot spots).

The case of LDH is presented here as a paradigmatic example to illustrate the insight offered by our method. As a comparison, we first compute all normal modes (NM) by brute-force diagonalization of the dynamical matrix. The patterns of the highest-frequency NMs (Fig. 2A) reveal that they are highly confined to some very specific residues. We then compute the high-frequency LL of the enzyme (indicated by *u* in Fig. 2B). The most interesting property of the LL appears when comparing it to the catalytic structure of the enzyme, characterized by the locations of the known active sites of LDH (VAL-31, GLY-32, MET-33, LEU-65, GLN-66). Clearly, catalysis in LDH takes place in the regions where the fast vibrations of amino acids are preferentially concentrated. The same analyses repeated for the biological assembly of LDH (tetramer) yield the same results (see Supplementary Information), indicating that the dimer-dimer interface does not harbor vibrational modes specifically associated with the enzyme activity.

**Figure 3:**
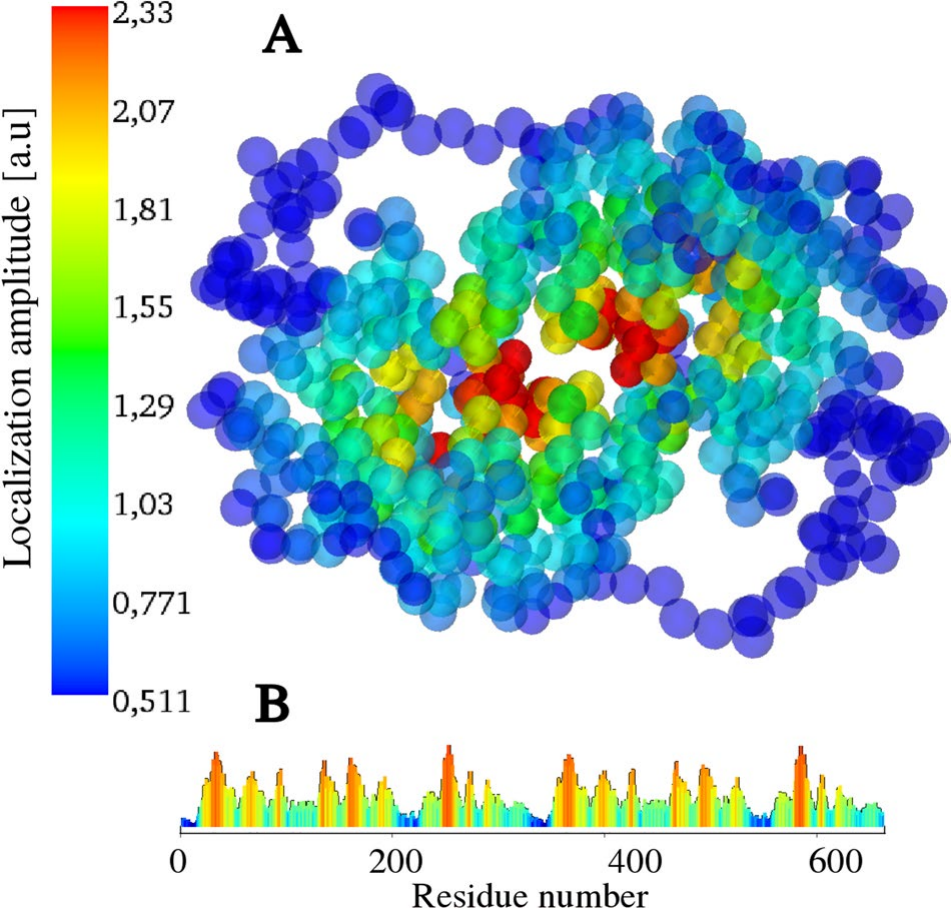
3D LL for human L-Lactate Dehydrogenase dimer. The 3D LL is shown by color-coding the 3D coarse-grained scaffold according to the amplitudes of the LL depicted in Fig. 2B. We observe here that peaks (hot spots) of the localization landscape that appear distant when plotted along the backbone chain are in fact found around the same spatial location (the red spots). Wave localization is thus predicted to occur within two distinct 3D domains lying at the center of the molecule: these domains host the catalytic activity.

The structure of the localization pattern appears even more clearly when color-coding it onto the 3D conformations (Fig. 3), thus identifying unmistakably two distinct regions in the molecule where fast vibrations are concentrated. We observe here that peaks (hot spots) of the localization landscape that appear distant when plotted along the backbone chain (Fig. 2B) are found around the same spatial locations (here, the two red spots in Fig. 3). We also find that the few peaks of the localization landscape that do not seem to correspond to any active site are in fact found in the same regions, once the backbone chain is folded into its tertiary structure. This observation applies very generally to all LLs computed for a very large set of enzymes (see Fig. 7 hereafter).

**Figure 4:**
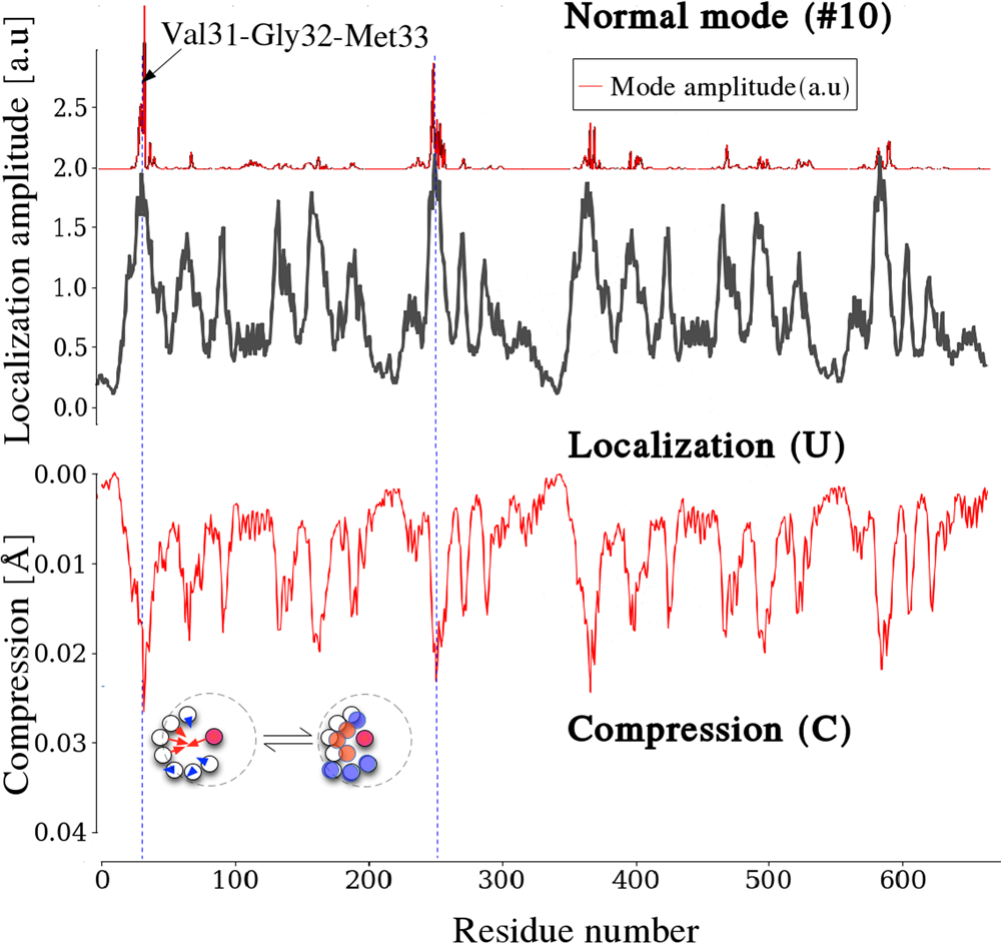
Compressive motions and localization sites in the L-Lactate Dehydrogenase dimer (LDH). The displacement amplitude (top graph) associated with the vibrational eigenvector #10 (frequency 94.17 cm^−1^, 2.82 THz) is localized along the reaction coordinate residues VAL-31, GLY-32, MET-33, as predicted by the LL (function **U**, middle graph). The computation of the local compression factor (see Supplementary Information) clearly shows that these localized modes are compression modes.

**Figure 5:**
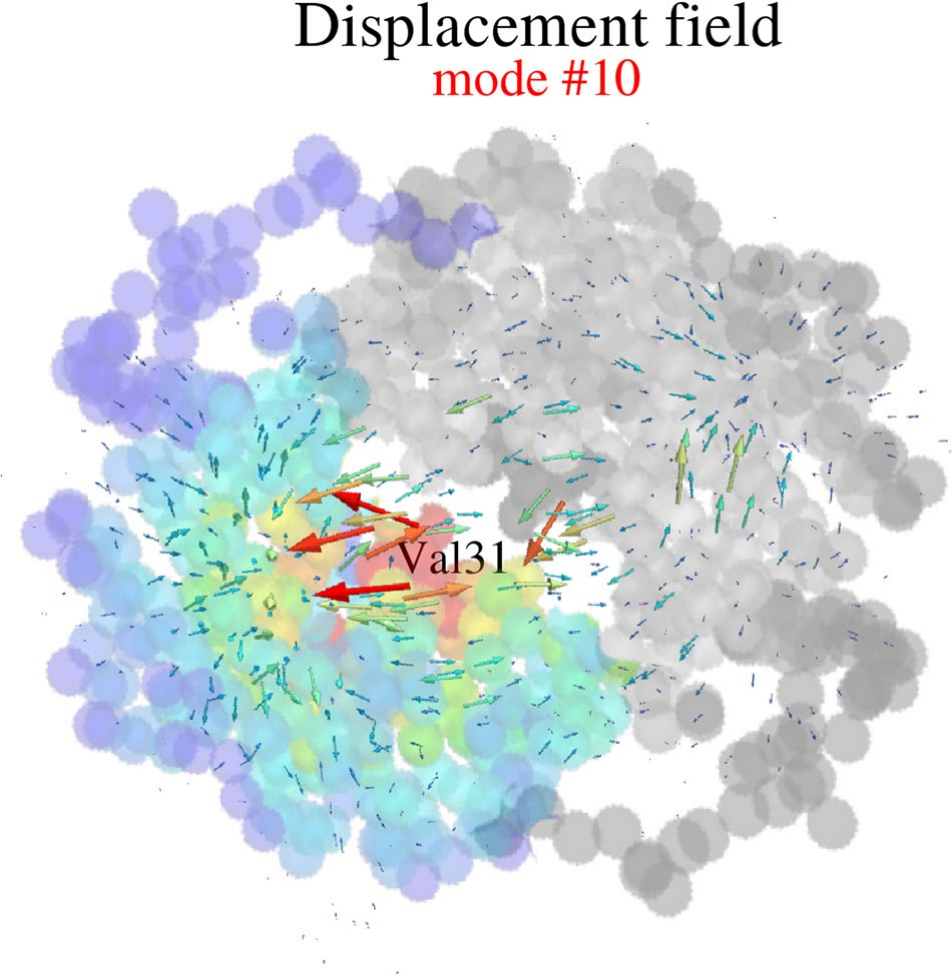
A rate-promoting vibration in the L-Lactate Dehydrogenase dimer (LDH). Localization landscape color-coded on the coarse-grained structure with a close-up of the compression field corresponding to the vibrational eigenvector #10 (frequency 94.17 cm^−1^, 2.82 THz) along the reaction coordinate: residues VAL-31, GLY-32, MET-33 compress towards ARG 106. The localized eigenmode #10 corresponds to the rate-promoting vibrations found in ref.^19^.

A careful analysis of the spatial structure of localized modes reveals that high-frequency localized vibrations are *compressive* motions (see Supplementary Information). Hence, at hot-spots, amino acids tend to get close-packed. This feature is demonstrated here by computing at each residue the reduction of the mean distance between nearest neighbors induced by the highest-frequency modes. Figure 4 displays the result of this computation in the case of LDH: we clearly see that localization hot spots match almost exactly the regions subjected to compression motions of large magnitude. A more detailed analysis of a localized mode is presented in Fig. 5 (the example shown in the figure is the eigenvector #10).

**Figure 6:**
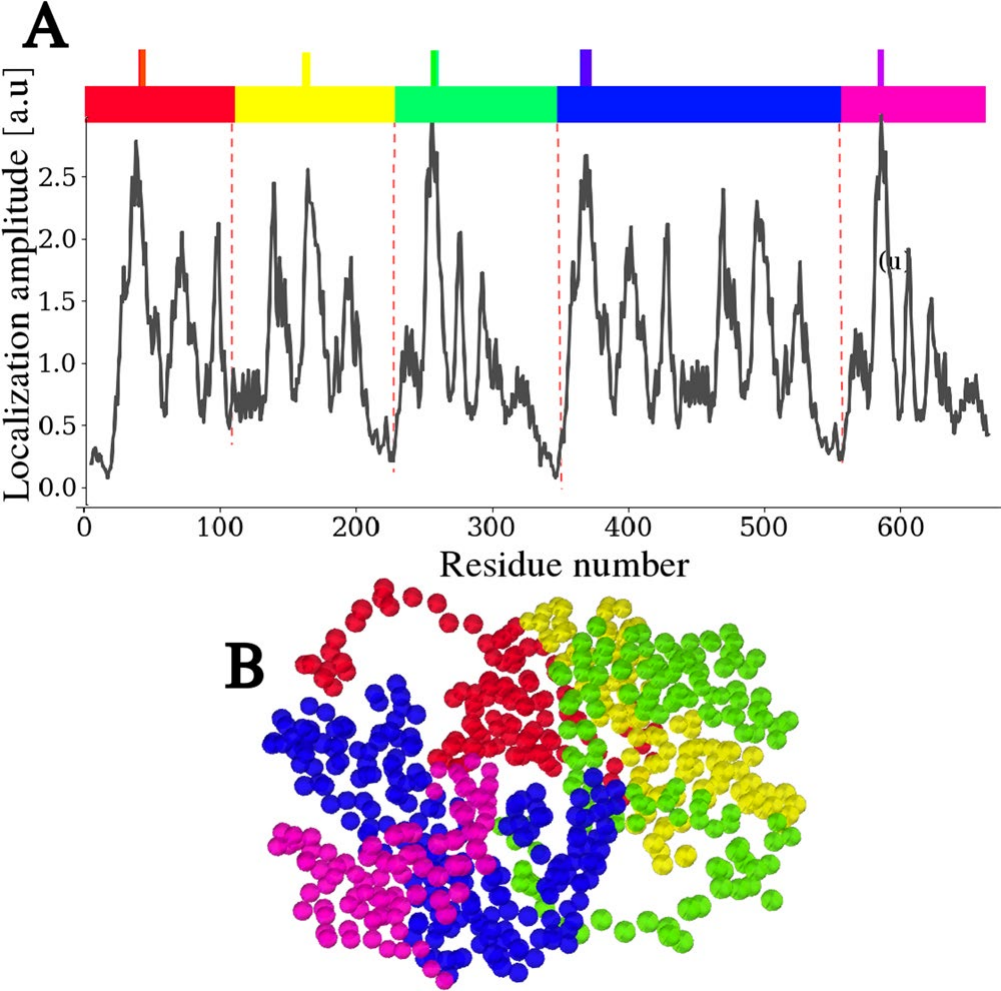
Localization and functional domains. (**A**) Partitioning of the molecule obtained from the LL. On the landscape plotted on the backbone chain, one selects the 4 highest local maxima (marked by a spike on the color bar) separated by the 4 lowest local minima (marked by the dotted lines). In the LL theory, each domain can be seen as a local harmonic oscillator, weakly coupled to the others. (**B**) The partitioning of the LDH obtained in frame A is here plotted on the tertiary structure, exhibits distinct spatial domains.

An important additional feature revealed by the LL analysis of LDH is that the enzyme structure appears to be partitioned into large-scale domains, i.e., contiguous sets of sites separated by deep minima of the landscape function (Fig. 6A). These domains comprise few hundreds of amino acids associated with the oligomeric complexes (monomer, dimer, trimer etc.). Each of these domains exhibits a sub-structure comprising 2 to 4 regions of a few tens of sites that harbor the most localized vibrations. From the LL, we can define each domain as comprising a hot-spot and extending to the two lowest local minima on both sides along the chain. Each of them can be understood as a nearly independent vibrational region (see Fig. 6B), weakly coupled to its neighbors. This representation offers a totally new functional vision of the protein and also paves the way for a new understanding of allosteric processes^47^. This aspect will be addressed further in the Discussion section.

**Figure 7:**
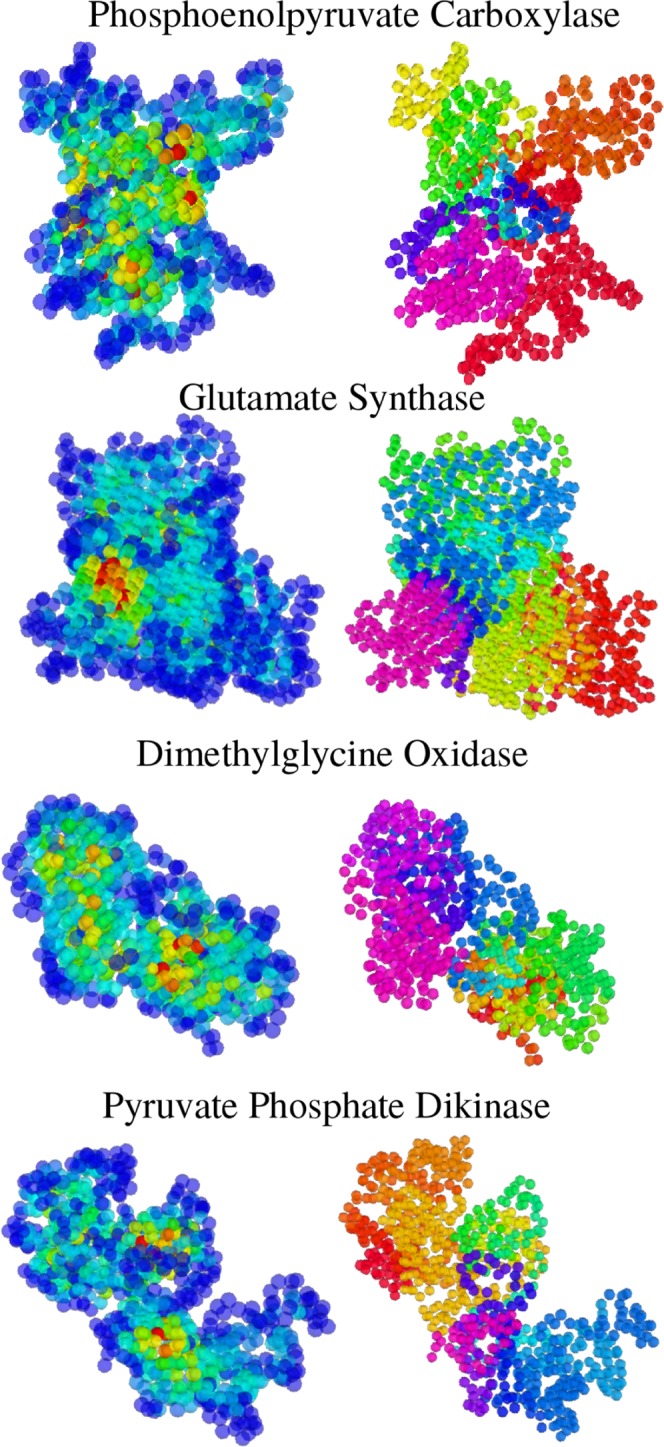
Domains in other enzymes. The partitioning procedure is illustrated for four enzymes. The clustering of the enzymes into vibrationally independent subregions is a general feature.

The subtle connection unveiled above between localization of vibrational energy and compressive reorganization of the active site is by no means an isolated case. This has emerged neatly from the systematic study of a set of 933 enzymes from the catalytic site atlas^48^, comprising a total of 10,566 experimentally annotated catalytic sites. For each enzyme, we have computed the LL and located its highest maxima (examples of 3D representations of LLs for several enzymes are displayed in Fig. 7, left column, while the right column displays the partitioning of each enzyme into independently vibrating domains, obtained from the LL using the procedure illustrated in Fig. 6).

**Figure 8:**
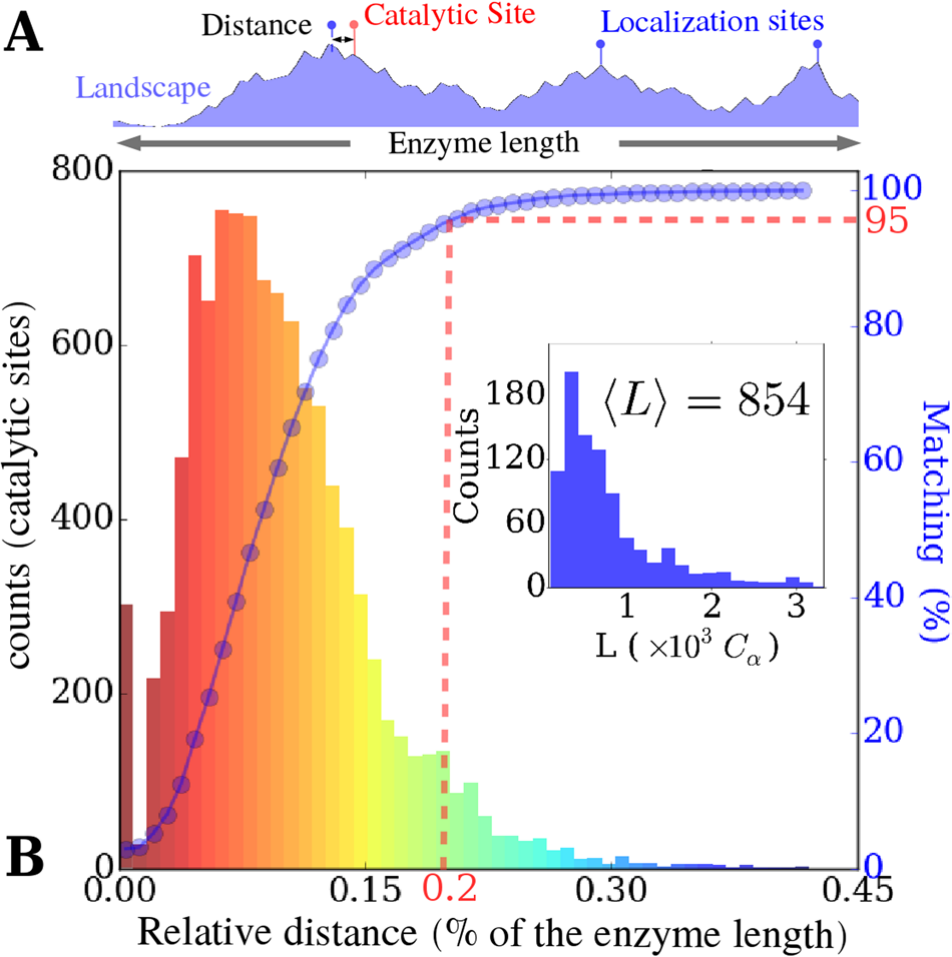
Proximity score for 10,566 annotated catalytic sites (933 enzymes) from the catalytic site atlas^48^, gauging the match between a functional site and a main localization hot spot. Frame A: The relative distance is scored by taking the shortest distance between catalytic sites and the main localization hot spots, divided by the chain length. Frame B: Main histogram. 95% of active sites are found at one of the highest localization spots with an error smaller that 0.2% of the enzyme length along the chain. Inset: Size distribution.

Then, for each known catalytic site of the enzyme, we have computed the distance to the nearest maximum of the LL, expressed as a percent of the total length of the backbone chain (see Fig. 8A). Figure 8B displays a histogram of these relative distances, computed over all enzymes and all catalytic sites. The dotted curve plotted on top of the histogram represents the cumulative score. In 95% of cases, a catalytic site is found within 0.2% of the total chain length from a localization hot spot. By comparison, the distance along the chain between a site picked at random and the nearest localized vibration site would be on average 10% of the chain length, i.e., about 200 times farther away! This striking concordance clearly indicates that vibrational energy localization, as dictated by the 3D scaffold, must play a key role in the design of enzyme function: in 95% of the case, catalytic sites are located in domains where residues exhibit fast compressive motions.

## Discussion

Localization of vibrations is a general feature of the scaffold of proteins. The LL is a novel theoretical tool that allows one to capture quickly and efficiently the fundamental relationship between the 3D structure and the spatial pattern of localized vibrations, firstly by predicting their locations and secondly by showing how the complex and irregular shape of the macromolecule can be partitioned (segmented) into a few weakly coupled clusters of vibrations. These highly localized vibrations involve few specific residues with periods of the order 2–4 ps, and systematically take the form of compressive motions. Channeling thermal (or non-equilibrium) vibrational energy along such specific localized eigenvectors could be crucial for optimal enzyme functioning, e.g. in reducing the transfer distance associated with transition-state barriers or modulating donor-acceptor distances along specific directions, thus accelerating the chemical reaction step. Our analysis through the LL, performed on 933 enzymes, has confirmed that the overwhelming majority of their catalytic sites are located at hot spots and are henceforth at the core of specific, fold-rooted compressive motions.

It should be stressed that the fact that catalytic sites tend to sit in mechanically rigid regions of enzyme structures is relatively well-known among structural biologists. The merit of quantitative studies such as Refs.^35,37^ was to reignite interest in this fact from the standpoint of the prediction of active sites. The present paper reports fundamental hints as to why those methods work, unveiling a hitherto unknown deep interconnection between wave localization in complex 3D enzyme structures and their catalytic function. Our main claim is that enzyme structures have been so crafted by evolution so as to imprint a unique interference pattern to thermal fluctuations, featuring transient hot-spot patterns and substantial channeling along specific compressive motions at the active site. These modes, e.g. coupling directly to the donor-acceptor distance in H-transfer-catalyzing enzymes, are decisive in promoting the catalytic step. Our results not only draw a fundamental link between the structure of an enzyme and its vibrational energy pattern, they do so by tracing this back to a general fundamental^44,49^ physical quantity called the localization landscape (LL).

Incidentally, the highly specific dynamic modulations occurring at hot-spot sites and encoded in the LL structure are likely to make tunneling a viable pathway for H-transfer reactions at room temperature^22^. These considerations can be given proper physical meaning in the context of a phenomenological modified Marcus-like tunneling theory that is used with success to interpret experimental data on such enzyme-catalyzed reactions^50^. According to this theoretical scheme, the overall tunneling rate can be written as

1 $${k}_{t}\propto {e}^{-\beta {(\Delta G+\lambda )}^{2}/4\lambda }\int {e}^{-{S}_{G}(R\mathrm{)/2}\hslash }\,{{\mathscr{P}}}_{e}(R)\,dR$$

In the above expression $\varDelta G$ denotes the free energy barrier associated with the global transition between reactant and product in the multi-dimensional space of heavy nuclear coordinate and *λ* is the corresponding reorganization energy, both associated with slow conformational sampling needed to reach the tunneling-ready state. The effect of rate-promoting vibrations is to weigh H tunneling from the ground-state, here expressed in the WKB approximation through the ground-state action ${S}_{G}(R)$ which is a function of the donor-acceptor distance *R*. The rate-promoting vibration(s) specifically couple to the donor-acceptor distance providing a slow modulation (compared to tunneling times) of the donor-acceptor potential energy represented by the equilibrium probability density ${{\mathscr{P}}}_{e}(R)$ corresponding to *optimal compression* through rate-promoting motions along the donor-acceptor distance at the active site. The characteristic times for thermally activated barrier crossing and/or tunneling in an enzymatic reaction are fast compared to the period of typical rate-promoting vibrations associated with the local reorganization of the active site (ps-ns), which are themselves swift compared to the time-scales of slow conformational sampling and conformational changes (ms-s). This hierarchy of time scales allows localized motions to slowly modulate (with respect to the actual transition step) the energy landscapes associated with chemical reactions. However, such modulations occur millions of times per second while the 3D conformation of the protein appears frozen, as the free energy landscape associated with the global reactant-product equilibrium is essentially static at the scale of the transition state lifetime.

The striking and universal correspondence between the enzymatic active sites and the localization hot spots strongly suggests that the ps-ns time-modulated compressions at hot spots are a basic feature of enzymes that is likely the product of evolutive optimization. More generally following Occam’s razor, it is natural to imagine that all proteins or protein attempts (in an evolutionary sense) would display hot-spots and the associated compression localized vibrations for a few specific sites. If a given structure (i.e. the associated vibrational localization landscape) came at a certain point to form a rate-promoting match with a specific substrate (chemistry would be major the driving force) in a given unoccupied or rate-limiting metabolic niche, it is highly likely that that protein would have been increasingly stabilized and we would interpret its sequence as that of an enzyme now and that specific hot spot as its active site. Incidentally, this argument also makes it perfectly logical that there might exist more hot-spots encoded in the LL of a given enzyme than (presently known) active sites. Interestingly, strong support for this simple line of reasoning comes from another level of information encoded in the localization landscape. We show that the LL provides a natural and essentially parameter-free way to partition a given structure into vibrationally independent domains. At the same time, this clearly identifies groups of residues that belong to the same segment, and hence can vibrate together with minimal resonant loss to the other domains, while possibly sitting in principle sit at very distant locations in the 3D structure. This sounds precisely as a simple, totally novel rationale for allosteric coupling which can thus be regarded as deeply connected to the localization fingerprint of the scaffold^51^. In the perspective of the LL theory, allosteric coupling may thus be understood as a resonant phenomenon involving distant regions belonging to the same subdomain, as dictated by the landscape. Remarkably, communication at a distance (allostery), once thought to be a distinctive characteristic of few specific multimeric proteins, is now believed to be a universal characteristic of large folded biomolecules^52^. Again, such an intrinsic quality of protein folds, as shaped by their steric and chemical constraints, is encoded uniquely in the localization landscape and its associated layout of hot-spots/compression motions.

In the light of the above arguments, it seems rather natural to expect that a given protein should feature more than one hot-spot and associated compression mode in general. Those could be binding sites related to metabolic pathways that came to a dead-end or even flag hitherto unknown active or allosteric sites. More generally, the recent discovery of biased/multiple signaling^53^ in G protein-coupled receptors offers an intriguing case in point. In essence, the allosteric response of a given membrane receptor, i.e. the dynamical and conformational rearrangements occurring in the intra-cellular domains upon hot-spot activation (ligand binding) in the extra-cellular region, can vary if different hot-spots are activated at the same time. This means that different signaling pathways can be selected based on a single receiver (the receptor) reading an instruction written in a many-letter alphabet (more than one ligand/hot-spot pair). Remarkably, the experimental confirmation that cell receptors possess a significantly higher capacity than 1 bit of information (i.e. on or off response) has just been published^54^. This means that many active (binding) sites are present in the extra-cellular region of the receptor, and that the dynamical rearrangements with the association of the respective ligands must be tightly connected with the distinctive interference pattern that governs wave localization in the protein scaffold. Again, this information is entirely encoded in the LL.

In summary, we have uncovered a mathematical structure (the localization landscape) underlying the pattern of hot spots of vibrational energy localization in proteins. In the case of enzymes, those are invariably associated with active sites and the associated compression modes match known rate-promoting vibrations in the 50–300 cm^−1^, i.e., 1.5–9 THz, range^34,55^.

Importantly, we have exposed for the first time a deep connection between wave localization and long-range communication in proteins: the localization fingerprint encoded in the LL dictates in general the way a given protein scaffold is intrinsically partitioned into vibrationally independent domains that can span distances comparable with the protein size. Residues are thus resonantly tightly coupled within the same domain (even if sitting at large separation) with minimal leakage of vibrational energy to other domains.

The localization landscape theory has allowed us to unveil the simple, yet powerful building rationale behind the sophisticated evolutionary design of amino acid-based folded assemblies. It appears from our theory that a given 3D folded polypeptide chain is naturally endowed with two basic features, namely (i) a unique pattern of local compression modes at few hot-spot sites and (ii) an associated partition of the 3D structure into large, fold-spanning independent domains. The localization landscape is the one function that encodes this two-fold fundamental information.

## Supplementary information

Supplementary Information
