# Supplementary material for: Universality of fold-encoded localized vibrations in enzymes
Source: Sci Rep. 2019 Sep 6;9:12835. doi: 10.1038/s41598-019-48905-8 (PMC6731342; doi:10.1038/s41598-019-48905-8)
Supplement: Supplementary file 1 — Supplementary Information [file 41598_2019_48905_MOESM1_ESM.pdf]

Supplementary Information.
Universality of fold-encoded localized vibrations in enzymes.

Yann Chalopin,[1,*] Francesco Piazza,[2] Svitlana Mayboroda,[3] Claude Weisbuch,[4,5] and Marcel Filoche[4]

[1] *Laboratoire d'Energétique Macroscopique et Moléculaire, Combustion (EM2C), CentraleSupélec, CNRS, 91190 Gif-sur-Yvette, France*
[2] *Centre de Biophysique Moléculaire (CBM) CNRS UPR4301 & Université d'Orléans, Orléans 45071, France*
[3] *School of Mathematics, University of Minnesota, Minneapolis, Minnesota 55455, USA*
[4] *Laboratoire de Physique de la Matière Condensée, Ecole Polytechnique, CNRS, 91128 Palaiseau, France*
[5] *Materials Department, University of California, Santa Barbara, California 93106, USA*

I. ELASTIC NETWORK MODEL OF PROTEIN DYNAMICS

Elastic network models (ENM) of protein dynamics have been introduced by M. Tirion in 1996 [1] and later reformulated in a coarse-grained version by Bahar and coworkers under the name of anisotropic network model (ANM) [2]. In the ANM, a given protein comprising N residues is represented by an ensemble of N fictitious particles, the mass of each particle being concentrated at the location of the corresponding α-carbons. By definition, the equilibrium configuration of the system is taken to coincide with the experimentally solved structure (i.e., from X-ray diffraction or as an average over several NMR conformers). All particles are taken to have the same mass, which we set equal to the average amino acid mass $M = 110$ a.m.u., and each particle interacts with its neighboring particles through a central harmonic force. Let us denote $\boldsymbol{r}_i(t)$ and $\boldsymbol{R}_i$ the instantaneous and the equilibrium position vector of the i-th residue, respectively. The total potential energy of the system is that of a network of beads and central springs, that is,

$$V = \frac{1}{2}\sum_{i>j} K_{ij}(r_{ij} - R_{ij})^2\,, \tag{1}$$

where K_{ij} is the force constant of the spring connecting the residues i and j, while $r_{ij} = |\boldsymbol{r}_i - \boldsymbol{r}_j|$ and $R_{ij} = |\boldsymbol{R}_i - \boldsymbol{R}_j|$ are the instantaneous and equilibrium Euclidean distances between the pair (i, j). The matrix of force constants can be specified in several ways. Here, in line with the original ideas of the ENM modeling strategy, we use a single stiffness k for all springs and identify the set of interacting pairs through a connectivity matrix, that is,

$$K_{ij} = k\, c_{ij} \tag{2}$$

where $c_{ij} = \{1 \text{ for } R_{ij} \leq R_c \text{ and } 0 \text{ otherwise}\}$. According to previous studies [3], we set $k = 5$ kcal/mol/Å^2 and choose a cutoff $R_c = 10$ Å. In order to compute the localization landscape of a protein, we consider the harmonic approximation of the ANM, which corresponds to

$$V = \frac{1}{2}\sum_{ij}\sum_{\alpha\beta} \mathbb{H}_{ij}^{\alpha\beta} u_{i\alpha} u_{j\beta} + \mathcal{O}(\|u\|^3) \tag{3}$$

where $u_{i\alpha} = r_{i\alpha} - R_{i\alpha}$ $(\alpha = x, y, z)$ are the Cartesian components of the displacement vector of residue i. The Hessian matrix $\mathbb{H}$ is directly derived from the total potential energy through

$$\begin{aligned}\mathbb{H}_{ij}^{\alpha\beta} &\stackrel{\text{def}}{=} \left.\frac{\partial^2 V}{\partial u_{i\alpha}\partial u_{j\beta}}\right|_{\{u=0\}} \\ &= -K_{ij} s_{ij}^{\alpha} s_{ij}^{\beta} + \delta_{ij}\sum_m K_{jm} s_{mj}^{\alpha} s_{mj}^{\beta}\end{aligned} \tag{4}$$

where $s_{ij}^{\alpha} = R_{ij}^{\alpha}/R_{ij}$ are the Cartesian components of the unit equilibrium inter-particle vectors. The normal modes (NM) of a system of interacting particles, such as the residues in an elastic network, are the eigenvectors of the mass-weighted Hessian matrix (also known as dynamical matrix),

$$\widetilde{\mathbb{H}} = M^{-1/2}\ \mathbb{H}\ M^{-1/2} \tag{5}$$

where M is the diagonal mass matrix. It is well known that the high-frequency NMs of vibrations of protein structures are strongly localized in space, which is a result of the spatial quenched disorder of their equilibrium structures [2]. This is still true in our coarse-grained model where the highest frequencies are of the order of 100 cm^{-1} (3 THz) and the corresponding displacement vector fields are localized in regions of the size of one coordination shell, i.e., $\mathcal{O}(R_c)$.

II. THE LOCALIZATION LANDSCAPE OF THERMAL PHONONS

A. Calculation of the localization landscape

Within the ANM framework, the equations of motion read

$$M_i \ddot{u}_{i\alpha} = -\sum_{j\beta} \mathbb{H}_{ij}^{\alpha\beta} u_{j\beta} \tag{6}$$

* Correspondence to yann.chalopin@centralesupelec.fr

By introducing the mass-weighted coordinates $X_{i\alpha} = \sqrt{M_i}\; u_{i\alpha}$, this set of equations can be put into the following vector form:

$$\ddot{\mathbf{X}} = -\widetilde{\mathbb{H}}\,\mathbf{X} \tag{7}$$

We look for solutions to Eq. (7) in the form $\mathbf{X} = \mathbf{Y}e^{-j\omega t}$, which amounts to solving the related eigenvalue problem, i.e., finding the eigenvectors $\mathbf{Y}^n$ and frequencies ω_n such that

$$\widetilde{\mathbb{H}}\,\mathbf{Y}^n = \omega_n^2\;\mathbf{Y}^n \tag{8}$$

The displacement of residue i can be decomposed into the contributions along each eigenvector $\mathbf{Y}^n$, that is,

$$u_{i\alpha}(t) = \frac{X_{i\alpha}(t)}{\sqrt{M_i}} = \frac{1}{\sqrt{M_i}}\sum_{n=1}^{3N}\alpha_n Y_{i\alpha}^n\; e^{-j\omega_n t}. \tag{9}$$

Ref. [4] introduces a mathematical function called localization landscape (LL) for predicting low-frequency localization. Yet, in the case of an inhomogeneous discrete system, high-frequency eigenvectors also correspond to localized, short-wavelength vibrations. According to a procedure similar to the one developed in [5], a high-frequency LL can also be computed as the solution $\mathbf{U}$ to the following linear system

$$\widetilde{\mathbb{H}}_c\;\mathbf{U} = \mathbf{1}\,, \tag{10}$$

where

$$\widetilde{\mathbb{H}}_{c,ij}^{\alpha\beta} = \begin{cases} c - \widetilde{\mathbb{H}}_{ij}^{\alpha\beta} & \text{if } i = j, \alpha = \beta \\ \widetilde{\mathbb{H}}_{ij}^{\alpha\beta} & \text{otherwise.} \end{cases} \tag{11}$$

Here, c is a small real positive constant such that all eigenvalues of the matrix $\widetilde{\mathbb{H}}_c$ are positive. The physical idea behind this (see Ref. 5) is to look for localized modes of wave vector close to $k = \pi/a$ where $a \simeq 3.83$ Å is the equilibrium distance between consecutive α-carbons along the protein primary structure. This is the only 1D path belonging to the connectivity graph that ensures translational invariance along the chain. Finally, the localization landscape $\mathcal{U}$ used in the main paper above to exhibit the location of catalytic sites in enzymes is defined as the geometrical average of the three Cartesian components of $\mathbf{U}$, namely

$$\mathcal{U}_i = \left(\sum_{\alpha\in x,y,z} U_{i\alpha}U_{i\alpha}\right)^{1/2} \tag{12}$$

For sake of simplicity, we use the letter $\mathbf{U}$ to designate the localization landscape in the main paper.

III. COMPUTING EFFICIENCY OF THE METHOD

Another important aspect of this approach is its remarkable computational efficiency. The study of proteins motions is usually conducted through an analysis of the normal modes. This requires solving the eigenvalue problem (see Eq. (8) in Appendix II)

$$\widetilde{\mathbb{H}}\,\mathbf{Y}^n = \omega_n^2\;\mathbf{Y}^n \tag{13}$$

where $\mathbf{Y}$ and ω_n^2 correspond to the normal modes and eigenfrequencies, respectively. Retrieving these quantities from normal modes analysis (NMA) can be a computational issue for large macromolecules (number of residues $N > 10000$), especially when long range interactions are accounted for, as they considerably reduce the sparsity of the matrix $\widetilde{\mathbb{H}}$. By contrast, the localization landscape is obtained by solving a simple linear system of algebraic equations

$$\hat{L}\mathbf{U} = \mathbf{1}, \tag{14}$$

where $\hat{L}$ stands for a self-adjoint operator constructed from the dynamical matrix (see Eq. (10) in Appendix II). Table I compares the computational cost of the two aforementioned approaches, by reporting the required CPU-time as a function of the number of degrees of freedom (d.o.f). The ratio between the CPU times required by the two methods is displayed in the last column. The LL approach is roughly 50 times more efficient for the typical protein size encountered in this study, although we have restricted this analysis to the case of tridiagonal matrices: in practice, the computational gap between the two methods is even more substantial in realistic systems. This performance offers a clear advantage for a systematic analysis of large sets of protein data.

TABLE I. Comparison between Normal modes (NMA) and localization landscape (LL) analyses.

	CPU time [s]		
# of d.o.f.	NMA	LL	Ratio NMA/LL
500	0.72	0.0032	22
1000	4.6	0.17	27
2000	40	1	40
5000	840	18	47
10000	6600	132	50
20000	54000	571	100

IV. CALCULATION ON THE BIOLOGICAL ASSEMBLY OF LDH.

The localization landscape has been calculated for the biological assembly of LDH. Figure 2 compares the dimer and the tetramer landscapes. Both localization patterns exhibit a similar structure, most of the dominant localization hot-spots and catalytic sites are found with the same proximity. Interestingly, this reveals that the interface between the two dimers does not play any significant role in the fast dynamical properties, localized vibrations at the distant catalytic sites remain unchanged.

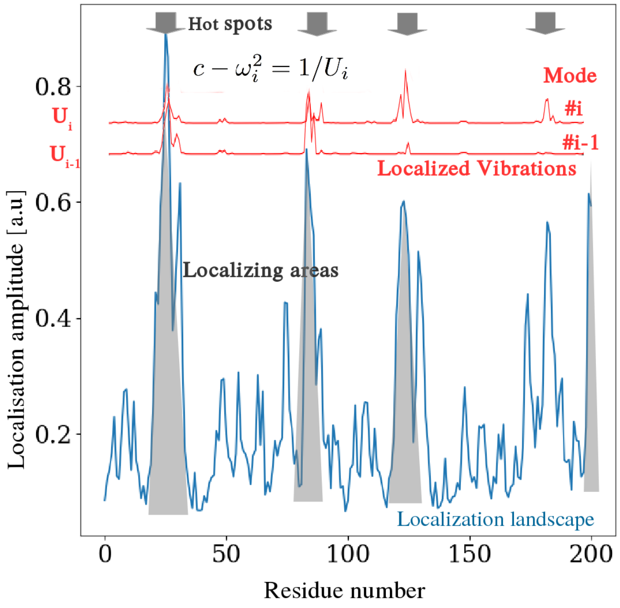

FIG. 1. **Localization Landscape for HIV-1 Protease (PDB id: 1A30)**. Wave localization is visualized through the displacement pattern of the 2 fastest eigenmodes (1 and 2), with frequencies of the order of 96 cm^{-1} (2.88 THz). The locations of the maxima identify the areas of strongest localization, i.e., the "hot spots". Each eigenfrequency can be associated with a peak height in the landscape, whose values depend on the choice of the constant c in Eq. (10). In this case, we have chosen $c = 18$ in non-dimensional units ($k = M = 1$), so that, in particular, all eigenvalues of the operator (10) are positive as desired. The maximum value of U (≈ 0.9) yields $\omega_{\max} = \sqrt{c - 1/U_{\max}} \approx 4.11$. With the choice $k = 5$ kcal/mol/Å^2, $M = 110$ a.m.u., this gives $\omega_{\max} \approx 94.8$ cm^{-1} (2.84 THz), in agreement with the maximum frequency found by brute-force diagonalization of the dynamical matrix. The line that cuts horizontally the landscape at a given height reveals where the vibrations at that particular frequency are observed along the backbone chain.

V. CALCULATION OF THE LOCAL COMPRESSION FACTOR

The compression factor $\mathcal{C}_i$ measures the average level of local compression at a given site. For a given pair i, j, this amounts to evaluating the change in Euclidean distance along a given normal mode with respect to the equilibrium distance R_{ij}. In mathematical terms, $\mathcal{C}_i$ reads

$$\mathcal{C}_i = \frac{1}{N_{\mathcal{S}}\, c_i} \sum_{n \in \mathcal{S}} \sum_j c_{ij} \left[R_{ij} - \left(\sum_{\alpha=x,y,z} (R_{ij}^{\alpha} + a(Y_{i\alpha}^n - Y_{j\alpha}^n))^2 \right)^{1/2} \right], \quad (15)$$

where $\mathcal{S}$ is the set comprising the $N_{\mathcal{S}}$ highest-frequency normal modes, $c_i = \sum_j c_{ij}$ is the connectivity of residue i and a is an arbitrary displacement in Å. In our calculation we chose $a = 1$ Å, smaller than half the shortest inter-residue distance $R_{ij} \simeq 3.8$ Å. This ensures that $\mathcal{C}_i$ are positive quantities, in agreement with the physical requirement that relative displacements cannot exceed equilibrium inter-distances.

[1] M. M. Tirion, Phys. Rev. Lett. **77**, 1905 (1996).
[2] I. Bahar and Q. Cui, *Normal Mode Analysis: Theory and Applications to Biological and Chemical Systems*, edited by B. R. CRC Press, Mathematical & Computational Bi-

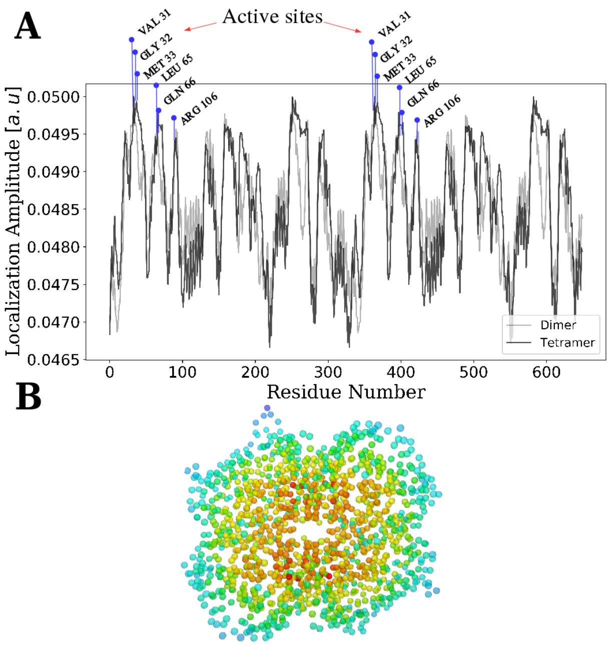

FIG. 2. **Localization Landscape for the biological assembly (tetramer)**. A: The localization landscape of the biological assembly (black) is compared to that of the dimer (grey). The catalytic sites are located on the same hot-spots. B: The localization landscape in 3D reveals that the localization domains of the biological assembly lie in the core region, as observed for the dimer.

ology Series, Vol. 9 (CRC Press, 2005).

[3] B. Juanico, Y.-H. Sanejouand, F. Piazza, and P. De Los Rios, Phys. Rev. Lett. **99**, 238104 (2007).

[4] M. Filoche and S. Mayboroda, Proc. Nat. Acad. Sci. USA **109**, 14761 (2012).

[5] M. L. Lyra, S. Mayboroda, and M. Filoche, EPL (Europhysics Letters) **109**, 47001 (2015).
